# Associations between self-efficacy, bullying and health-related quality of life in a school sample of adolescents: a cross-sectional study

**DOI:** 10.1186/s12889-019-7115-4

**Published:** 2019-06-14

**Authors:** Kristin Haraldstad, Lisbeth G. Kvarme, Knut-Andreas Christophersen, Sølvi Helseth

**Affiliations:** 1 Faculty of Health- and Sport Sciences, University of Agder, P.O box 422, 4604 Kristiansand, Norway; 2 Faculty of Health, OsloMet, Oslo Metropolitan University, P.O box 4, St Olavs Plass, 0130 Oslo, Norway; 3 Faculty of Social Sciences, University of Oslo, P.O box 1084, Blindern, 0317 Oslo, Norway

**Keywords:** Health-related quality of life, Adolescents, Self-efficacy, Bullying

## Abstract

**Background:**

To better understand health-related quality of life (HRQOL) in adolescents, it is important to gain knowledge about factors associated with HRQOL. Being involved in bullying is a significant threat to health, and social and psychological well-being; further, such problems can last into adulthood. The aim of this study was to explore the role of general self-efficacy (GSE) and bullying in relation to HRQOL. We specifically sought to study the prevalence of bullying, as well as the associations between both bullying and self-efficacy and HRQOL in a sample of adolescents.

**Methods:**

This was a cross-sectional study of 723 adolescents (12–18 years) attending schools selected using randomized cluster sampling. HRQOL was measured using the KIDSCREEN-52, self-efficacy was measured with the GSE scale, and bullying was measured using the two global questions from the Olweus bullying questionnaire. Multiple regression analyses were performed to explore how being bullied, bullying, and GSE were associated with variations in self-reported HRQOL.

**Results:**

Of the 723 adolescents, 13% reported being bullied; there were no gender differences within this finding. However, more boys than girls reported that they had bullied others. Both being bullied, and bullying others, were associated with lower HRQOL; however, being bullied was associated with the lowest scores. Higher self-efficacy was associated with better HRQOL. Self-efficacy contributed significantly to predicting variation in HRQOL.

**Conclusions:**

Being involved in bullying, as a victim or a bully, is associated with lower HRQOL. The association between GSE and HRQOL indicates that self-efficacy might be a resource for increasing HRQOL among adolescents. Our findings highlight the importance of targeting self-efficacy beliefs as an intervention strategy to improve GSE and HRQOL in adolescents involved in bullying.

## Background

The concept of health-related quality of life (HRQOL) as a general outcome is of growing interest in the field of public health and is acknowledged as a useful measure of health and well-being in both adult and adolescents populations [1, 2]. HRQOL is a multidimensional construct and includes measures of physical symptoms, functional status, psychological and social functioning [3]. The multidimensionality of health-related quality of life measures (HRQOL) gives researchers and clinicians information about the impact of a health condition, or the effect of various interventions on different aspects of HRQOL, and serves as a framework for identifying and developing strategies to promote HRQOL [2].

To enhance well-being among adolescents, and to better understand HRQOL in adolescents, it is important to study factors associated with HRQOL. HRQOL is a positive phenomenon and is therefore particularly relevant within the health promotion perspective, insofar as it focuses on resources rather than problems [4, 5]. The concept of self-efficacy has been suggested as one such focus. Self-efficacy is a central part of Bandura’s [6, 7] social cognitive theory. Self-efficacy includes individuals’ thoughts about their successes and failures, and their perceptions of feedback they have received. Previous studies have shown that a high degree of self-efficacy is related to higher self-belief [8], and that self-efficacy is one of the most important factors contributing to behavior change [9]. This concept was expanded upon by Schwarzer and Renner, who developed the term general self-efficacy (GSE), which is used herein [10].

Positive associations have been reported between GSE measures and HRQOL among adults, but few studies have examined this in children or adolescents [5]. Among those that have, Kvarme and colleagues [5] reported a strong, positive relationship between GSE and HRQOL among schoolchildren, indicating that GSE might be a resource for increasing HRQOL in this population.

Though bullying is a factor known to negatively affect adolescents’ health and well-being, few studies have regarded the associations between bullying and HRQOL. A relatively small number of studies do show that being bullied by peers, and bullying others, are both significantly related to lower HRQOL levels [5, 11–17]. The majority of these studies have shown that being involved in bullying is related to lower HRQOL, particularly on scales measuring social and mental health [11, 17].

In recent years, awareness of bullying among adolescents has increased and is now widely recognized as one of the most significant public health problems within this age group [4]. In a systematic review, a mean prevalence of bullying was reported to be 35% and victimization was found in 36% of adolescents aged 12–18 years [18]. However, the prevalence of bullying differs across studies [5, 19–21], with some studies showing a prevalence in Norway and the other Scandinavian countries that is slightly lower compared with most other countries [22–24].

Several studies have shown that being bullied is a significant threat to both short- and long-term health, as well as social and psychological well-being. Those who are bullied during childhood are more likely to report depression, social anxiety low self-esteem, and academic problems later in life [11, 25–29]. In addition, bullies themselves can have long-term health challenges, including a high prevalence of antisocial problems, physical health problems, and higher use of health support [30–34].

The most common definition of bullying is that a person is bullied “when she or he is exposed to negative actions from one or more persons recurrently over time” [35]. A negative action is when a person intentionally inflicts, or attempts to inflict, discomfort or injury upon another person. Bullying occurs in a relationship in which there is an imbalance of power or strength [36, 37].

Because bullying is complex, it is important to study this problem from different perspectives. Most previous studies have explored the negative health consequences related to being bullied. From a health promotion perspective, it is important to study how this phenomenon is related to adolescents’ HRQOL [38]. Although much of the research in this area has focused on understanding bullying and its health consequences, far less is known about the associations between bullying, self-efficacy, and HRQOL in adolescents.

The aim of this study was therefore to explore the roles of self-efficacy and bullying in relation to HRQOL. We specifically sought to determine the prevalence of bullying and examine the associations between both bullying and self-efficacy and HRQOL in a sample of adolescents.

## Methods

These cross-sectional analyses are part of a larger study of HRQOL among Norwegian adolescents in an eastern region of Norway with a population of approximately 1.7 million, among which the child/adolescent population is approximately 230,000. For this study, Statistics Norway drew a cluster sample of 19 randomly selected schools using the criteria: geographic spread, rural and urban districts, and small and large schools. The schools were each sent a letter of invitation, which was followed by a telephone call. The seven schools that did not elect to participate were substituted with other schools selected based on the same criteria. From the final sample of 19 schools, classes that covered grades 7 and 9 in elementary schools, and grades 1 and 3 in secondary schools were selected; the age range included was thus 12–18 years.

After school enrollment in the study, the adolescent students were invited to participate. They and their teachers were also given verbal and written information at school by the investigator 1 week before the study took place. They received standard information about the study and a written consent form for the adolescents’ parents to read and sign. The self-report questionnaires were administered and completed in the classrooms during school time.

The study was reviewed and approved by the Regional Research Ethics Committee of Norway (REK. Sør S-06143).

## Instruments

### Demographic variables

The first part of the questionnaire recorded demographic details for nationality, gender, date of birth, cohabitant status, and parental marital status.

### Self-efficacy

Self-efficacy was measured with a GSE scale ranging from 1 to 4 [31]. The scale was designed to measure a general sense of perceived self-efficacy and aimed to predict an ability to cope with daily demands and adaptation after a stressful experience. A revised five-item version of the questionnaire, designed for the general population aged 12 years or older, was used for this study [39].

A questionnaire item example is, “I always manage to solve difficult problems if I try hard enough.” The instrument has a four-point scale where 1 = “completely wrong” and 4 = “completely right.” Higher scores indicate higher levels of GSE. This scale has been shown reliable and valid, with a Cronbach’s α between 0.75 and 0.90 [5, 10, 40].

### Bullying

Bullying was measured using two global questions [26, 35] that were developed in Norway and have been used in previous studies [29, 41]. Bullying herein was defined according to the definition by Olweus [37]. The two questions address how often the child had been a victim of bullying or has bullied others during the previous 3 months. For example, “How often have you been bullied?” and “How often have you bullied others?” Both questions were answered using a five-point scale where 1 = “never”, 2 = “only once or twice”, 3 = “two or three times a month”, 4 = “about once a week”, and 5 = “several times a week.” In the analyses presented here, the two bullying variables were dichotomized as *never* or *only once or twice or more.*

### Health-related quality of life

HRQOL was measured with the Norwegian version of the KIDSCREEN-52 [42]. KIDSCREEN-52 is a generic questionnaire developed to evaluate HRQOL in children and adolescents and includes dimensions such as family, social relationships, physical functioning, psychological well-being, self-perception, school, autonomy, and financial resources [2, 43]. The instrument provides a comprehensive indication of health and well-being in children and adolescents [44]. The instrument includes 52 items, each of which is rated on a five-point Likert scale referring to the last week. The scale indicates either the frequency of certain behaviors or feelings (where 1 = “never”, 2 = “seldom”, 3 = “sometimes”, 4 = “often”, and 5 = “always”) or the intensity of an attitude (where 1 = “not at all”, 2 = “slightly,”, 3 = “moderately”, 4 = “very”, and 5 = “extremely”). Though these 52 items are usually divided into 10 dimensions, we used only nine of these dimensions after removing the three questions about bullying. The following dimensions were used: (1) physical well-being (five items); (2) psychological well-being (six items); (3) moods and emotions (seven items); (4) self-perception (five items); (5) autonomy (five items); (6) parental relations (six items); (7) financial resources (three items); (8) social support and peers (six items); (9) school environment (six items). Example questions from each dimension are: “When you think of the last week… (1) …have you felt fit and well?”; (2) “…have you felt satisfied with your life?”; (3) “…have you felt lonely?”; (4) “…have you been happy with the way you are?”; (5) “…have you had enough time for yourself?”; (6) “…have you felt loved by your parents?”; (7) “…have you had enough money to do things with your friends?”; (8) “…have you had fun with your friends?”; and (9) “…have you enjoyed going to school?” The scale was reversed for negatively worded items and missing values were substituted with the mean of the nonmissing items. However, according to the KIDSCREEN manual [45], no score was computed if more than one item per scale was missing and the three-item scales required that all items be completed. The dimension score was then transformed linearly to a 0–100-point scale, with 100 and 0 indicating the best and worst HRQOL, respectively. The KIDSCREEN-52 tool has been translated into several languages and its cross-cultural comparability and psychometric properties have been found satisfactory in different language versions. The Norwegian child version has shown satisfactory validity and reliability [42].

### Statistical analysis

The analyses were conducted using IBM SPSS Statistics (version 24.0; IBM Corp. Armonk, NY, USA). Descriptive statistics are presented for all dependent and independent variables. Cronbach’s α was computed to assess the reliability for each HRQOL dimension and self-efficacy. Pearson’s product moment correlation was used to examine the relationships between each of the nine dimensions of the KIDSCREEN-52 scale and independent variables. To examine how each independent variable—being bullied, bullying, and self-efficacy were associated with HRQOL, multiple linear regression analyses were performed with each of the nine KIDSCREEN subscales as dependent variables. For each HRQOL dimension, four regression models were estimated.

In the first model, the theoretically most important independent variable, self-efficacy, was the only independent variable. In the second model, the variables bullied, and bullying were added to the model as independent variables. In the third model, interaction between self-efficacy and respectively bullying and bullying were added, and in the fourth model, gender and age were also included as control variables.

The models were analyzed, using standardized independent variables. Then, the regression coefficients could be compared and, in addition, the importance of multicollinearity due to interaction was reduced.

The most important information lies in the regression coefficients, the change from one model to the next in regression coefficients and in R^2^-adjusted. While R^2^-unadjusted always increase when a new variable is added to a model, R^2^-adjusted can also decrease indicating that the model may be miss-specified having at least one irrelevant variable or an inadequate functional form.

## Results

Table 1 shows the sample characteristics for all dependent and independent variables. The age range was 12–18 years, with a mean of 14.6 years. After excluding adolescents with missing values, the sample eligible for inclusion in analyses was *N* = 723 (54% girls), response rate 74%. Of the participating adolescents, 13% reported being bullied within the previous 3 months (Table 1). In addition, 13% of the sample (9.4% girls, 17.5% boys) reported that they had bullied others.

**Table 1:** Descriptive statistics for all variables, ks01–09 are 9 kidscreen dimensions, *N* = 723

Variables	Min	Max	Mean	SD	Skewness	Kurtosis	Alpha
ks01 (Phys well-being)	0	100	65.00	21.25	−0.30	− 0.65	.84
ks02 (Psych well-being)	8	100	75.78	18.25	−0.94	0.69	.89
ks03 (Moods/emotion)	18	100	81.42	14.71	−1.39	2.22	.86
ks04 (Self-perception)	0	100	70.19	20.78	−0.64	−0.25	.82
ks05 (Autonomy)	0	100	68.51	20.73	−0.58	− 0.14	.85
ks06 (Parents-relationship)	8	100	75.75	19.04	−0.80	0.23	.88
ks07 (Financial resources)	0	100	78.53	23.28	−1.13	0.75	.89
ks08 (Peers and social supp)	21	100	73.14	17.07	−0.47	−0.35	.83
ks09 (School environment)	0	100	62.39	19.21	−0.21	− 0.24	.87
Gender (girls = 0, boys = 1)	0	1	0.46	0.50	0.17	−1.98	
Age	12	18	14.6	2.15	0.31	−1.22	
Bullied	0	1	0.13	0.34	2.15	2.64	
Bullying	0	1	0.13	0.34	2.19	2.79	
Self efficacy	1.20	4.00	3.07	0.60	−0.31	−0.38	

All nine HRQOL dimensions had skewness values of ±1.5 and eight had kurtosis values of ±1 (Table 2), indicating that these variables are approximately normally distributed.

**Table 2:** Correlations. Pearson’s r for all variables, *N* = 723

	ks01	ks02	ks03	ks04	ks05	ks06	ks07	ks08	ks09	Gen	Age	Bulli	Bully
ks02 (Psych. well-being)	.51												
ks03 (Moods/emotions)	.38	.66											
ks04 (Self-perception)	.39	.49	.54										
ks05 (Autonomy)	.44	.51	.40	.42									
ks06 (Parents relationship)	.35	.59	.54	.48	.44								
ks07 (Financial resources)	.28	.30	.34	.31	.29	.40							
ks08 (Peers & social support)	.31	.49	.32	.20	.44	.39	.24						
ks09 (School environment)	.40	.54	.44	.39	.40	.52	.36	.34					
Gender	.16	.09	.14	.34	.15	.06	.06	−.12	−.06				
Age	−.22	−.19	−.16	−.22	−.31	−.15	.01	−.09	−.27	−.02			
Bullied	−.07	−.19	−.20	−.14	−.13	−.14	−.22	−.23	−.14	.01	−.11		
Bullying	−.03	−.08	−.11	−.06	−.03	−.11	−.11	−.08	−.21	.12	−.06	.30	
Self- efficacy	.35	.32	.29	.35	.27	.28	.31	.21	.26	.21	.04	−.17	.00

Results from the correlations analysis (Table 2) show that HRQOL decreased significantly with age and that girls had lower HRQOL compared with boys on most of the KIDSCREEN subscales, meaning that being a boy and being among the youngest in the age group were both positively associated with HRQOL. Both being bullied, and bullying others, were negatively correlated with the HRQOL dimensions. Moreover, higher GSE scores were associated with better HRQOL across all dimensions.

Cronbach’s α values for the HRQOL scales were sufficient, from .82 (scale 4) to .89 (scale 7).

Multiple linear regression analyses were performed with each of the nine KIDSCREEN subscales as dependent variables (Table 3). In model 1 self-efficacy was included in the model and had a significant (*p* < .01) effect on all nine HRQOL scales, but the greatest effect on scale 1. Moreover, in model 2, the variables being bullied, and bullying were included. Being bullied had a significant effect on four scales (scale 2, 3, 7, and 8) and bullying on just one (scale 9). These three variables predicted from 7.9% (scale 7) to 12.8% (scale 4) of the variance.

**Table 3:** Regression coefficients^a^, 9 kidscreen dimensions, significance level .01 (bold) and .05 (italic), *N* = 723

	DV1: Physical well-being				DV2: Psychological well-being				DV3: Moods and emotions				DV4: Self-perception				DV5: Autonomy			
	M1	M2	M3	M4	M1	M2	M3	M4	M1	M2	M3	M4	M1	M2	M3	M4	M1	M2	M3	M4
Constant	***65.50***	***65.00***	***65.13***	***65.13***	***75.78***	***75.78***	***75.95***	***75.96***	***81.42***	***81.42***	***81.38***	***81.39***	***70.19***	***70.19***	***70.34***	***70.34***	***68.51***	***68.51***	***68.42***	***68.42***
Selfeff	***7.51***	***7.51***	***7.56***	***7.23***	***5.82***	***5.45***	***5.51***	***5.46***	***4.26***	***3.92***	***3.96***	***3.72***	***7.34***	***7.11***	***7.18***	***5.99***	***5.67***	***5.38***	***5.47***	***5.16***
Bullied		0.05	0.38	−0.12		***−2.24***	*−1.80*	***−2.16***		***−2.03***	***−2.11***	***−2.38***		−1.38	−1.00	*−1.55*		*−1.71*	*−1.92*	***−2.58***
Bullying		−0.68	−0.79	−1.15		−0.78	−0.92	−1.09		−1.01	−0.98	*−1.22*		−0.81	−0.93	*− 176*		− 0.16	− 0.09	− 0.51
Interaction1			0.78	0.80			1.04	1.06			−0.23	− 0.22			0.88	0.88			−0.58	−0.56
Interaction2			−0.99	−0.63			*−1.38*	−1.10			−0.41	−0.22			*−1.38*	−1.07			−0.92	−0.43
Gender				***1.95***				0.59				***1.38***				***5.99***				***2.04***
Age				***−5.05***				***−3.83***				***−2.74***				***−4.99***				***−6.77***
R^2^-adj^b^	***.124***	***.122***	***.123***	***.185***	***.100***	***.118***	***.123***	***.165***	***.083***	***.109***	***.108***	***.149***	***.123***	***.128***	***.131***	***.267***	***.070***	***.078***	***.079***	***.193***
R^2^-Change^c^		.001	.003	***.064***		***.020***	*.007*	***.044***		***.029***	.001	***.043***		***.007***	***.005***	***.137***		.007	.004	***.115***
	DV6: Parents relationship				DV7: Financial resources				DV8: Peers & social support				DV9: School environment							
	M1	M2	M3	M4	M1	M2	M3	M4	M1	M2	M3	M4	M1	M2	M3	M4				
Constant	***75.75***	***75.75***	***75.84***	***75.84***	***78.53***	***78.53***	***78.60***	***78.60***	***73.14***	***73.14***	***73.08***	***73.08***	***62.39***	***62.39***	***62.46***	***62.46***				
Selfeff	***5.24***	***5.04***	***5.09***	***5.11***	***7.10***	***6.51***	***6.56***	***6.53***	***3.66***	***3.10***	***3.09***	***3.74***	***5.04***	***4.92***	***5.05***	***5.57***				
Bullied		−1.22	−0.99	−1.29		***−3.53***	***−3.34***	***−3.39***		***−3.34***	***−3.49***	***− 3.64***		−0.73	−0.54	−1.02				
Bullying		*−1.81*	*−1.88*	***−1.99***		−1.51	−1.56	−1.60		−0.42	−0.37	− 0.10		***−3.82***	***− 3.88***	***− 3.81***				
Interaction1			0.53	0.54			0.41	0.42			−0.37	− 0.35			0.37	0.41				
Interaction2			−0.90	− 0.66			−0.92	− 0.88			0.26	0.44			***−1.90***	*−1.46*				
Gender				0.17				0.22				***−2.87***				***−1.96***				
Age				***−3.21***				−0.48				***−2.04***				***−5.55***				
R^2^-adj^b^	***.074***	***.089***	***.089***	***.115***	***.092***	***.122***	***.121***	***.119***	***− 045***	***.083***	***.081***	***.119***	***.068***	***.111***	***.119***	***.208***				
R^2^-Change^c^		***.017***	.003	***.028***		***.032***	.002	.001		***.041***	.001	***.040***		***.046***	*.010*	***.091***				

^a^ The coefficients are comparable because the independent variables are standardised while the dependent variable are not

^b^ Significance-testing is based on unadjusted R^2^

^c^ Changes are based on adjusted R^2^, reduction indicates that the model is not quite appropriate, and significance-testing is based on unadjusted R^2^

In model 3, two interaction terms were entered, one between self-efficacy and being bullied, and one between self-efficacy and bullying. The interaction between self-efficacy and bullying is significant (*p* < .05) on three scales (2, 4 and 9) in model 3. When controlled for gender and age in model 4, only the coefficient for scale 9 remains significant. In addition, the change in R^2^-adjusted decreases for scale 3, 7 and 8 from model 2 to model 3. Therefore, the main conclusion for model 3 is that the impact from the interaction terms is very weak.

In model 4 gender and age were included as control variables in addition to the three variables and the interaction terms. Self-efficacy still had significant (*p* < .01) effects on all scales and being bullied on five (scales 2, 3, 5, 7, and 8) and bullying on two (scale 6 and 9). Gender and age increased the predicted variance. The predicted variance now varied from 11.5% (scale 6) to 26.7% (scale 4). The lowest change in predicted variance was for scale 7 and the highest was for scale 4.

## Discussion

The purpose of this study was to describe the prevalence of bullying and associations between both bullying and GSE and HRQOL in a sample of adolescents aged 12–18 years. A main finding was that both being bullied, and bullying others were associated with lower HRQOL, and that a higher GSE was associated with better HRQOL. Self-efficacy contributed significantly to predicting variation in all HRQOL dimensions among this sample.

We found that 13% of the sample reported having been bullied during the previous 3 months, and that there were no gender differences within this finding. However, more boys than girls reported that they had bullied others. In a recent review, 20–25% of adolescents were involved in bullying as victims, perpetrators, or both [46]. In a meta-analysis on bullying prevalence, a mean prevalence of 35% for traditional bullying (both perpetration and victimization roles) and 15% for cyberbullying involvement were estimated [18]. The results of this meta-analysis showed a higher prevalence than detected in our study. Previous research has also demonstrated that the Scandinavian countries report a lower prevalence of bullying compared with other countries [25]. Possible explanations of this prevalence are strong anti-bullying policies, and focus on school-based anti-bullying programs, as for instance Olweus -programmes, in in the Nordic countries, most in Sweden and Norway [22, 23, 37, 47]. However, the prevalence rates of bullying vary between studies, partly because of differences in methods and questionnaires, which makes it difficult to compare results [48].

HRQOL is one way to study children’s subjective perspectives on bullying and the impact it has on their lives, in addition to the known negative impacts on their health and well-being [13, 17, 31]. The results of our analysis show that being involved in bullying—both as a bully and as a victim—is negatively associated with HRQOL. Being bullied was most strongly associated with HRQOL on the dimensions of “social support and peers”, “financial resources”, “autonomy”, “moods and emotions”, and “psychological well-being.” The dimension “social support and peers” had the lowest correlation value; this dimension examines aspects related to relationships with friends, the extent to which the person experiences positive group feelings, and support by his/her peers [2]. Relationships with friends are important at this age, and problematic peer relations are associated with psychological and social problems during adolescence [49]. Previous research has found that having friends and feeling peer support are positive and can protect children from bullying, while social support may be a buffer against bullying [21, 27, 46, 50].

Our results indicate that victims of bullying have lower HRQOL. They are generally less happy, may be less satisfied with their relationships with friends, have a less positive perception of themselves, are less positive about going to school, and are less satisfied with the financial resources in their family. This is in accordance with previous research; in a study from 11 European countries, being bullied was associated with significantly poorer HRQOL on all dimensions measured with the KIDSCREEN-52. The strongest associations were with the dimension “moods and emotions” and the weakest were with “physical well-being” [43]. A Swedish study also found that adolescents who reported being bullied had lower scores on all HRQOL subscales, measured with the SF-36 [17]. Findings from an Australian study show that being bullied is associated with significantly poorer psychosocial QoL; however, in that study, the physical dimensions were not related to being bullied [11].

In our study, it is notable that being involved with bullying—either as a bully or as a victim—had a negative association with HRQOL, although being a victim of bullying had the strongest association. However, bullying others had the strongest negative association with the two behaviors in the subscale “school environment.” A lower score on this dimension indicates that the individual has negative feelings about school and is not doing well at school [43]. Few studies have investigated both bullying and being bullied in relation to HRQOL, yet our results are consistent with a study from Sweden showing that being involved in bullying, as a bully or victim, was associated with lower HRQOL [17]. One possible explanation for this is the stress experienced from being involved with bullying, in either role [23].

Interestingly, in our study, being bullied was associated with lower HRQOL on the dimension “financial resources.” This dimension examines whether the individual is satisfied with his/her financial resources and takes part in activities with friends [43, 51]. It is well known that high socioeconomic groups do better on a range of health outcomes, and previous studies have shown that schoolchildren from single-parent families and those with lower socioeconomic status had the highest odds for emotional symptoms and were less resilient to bullying [52].

Our findings are consistent with a study showing that high GSE is associated with a better HRQOL [12]. Self-efficacy contributed significantly to predict the variation in all HRQOL dimensions among this sample, especially for the subscales physical well-being and self-perception. These scales explore levels of physical activity and energy, whether the adolescent feels well physically, and general feelings about how satisfied the adolescent feels about him/herself [43]. For adolescents who are victims of bullying, self-efficacy may mediate the negative effects of being bullied. A study from Greece showed that strengthening young persons’ GSE can reduce the health consequences of being bullied [38]. GSE is a personal resource related to the belief that you have the competence to handle difficult or new tasks and situations [40]. An adolescent with a low score on the GSE belief scale may be more likely to set lower personal goals and to have lower self-esteem. In the context of bullying, the adolescent’s poor self- esteem may affect his/her perception of their situation. Internal resources, such as increased self-efficacy, may be central to overcoming the victim role [5, 53].

Self-esteem and self-efficacy may be related concepts; however, self-esteem refers to the person’s thoughts about their worth, whereas self-efficacy is about the person’s judgments about their unique abilities to act and cope [6, 54]. Self-perception, as used in the Kidscreen questionnaire, refers to how satisfied the adolescent feels about him/herself as well as his/her appearance [45].

According to Bandura [7, 55], self-efficacy is a concept that can change, such as through goal-setting counseling, motivation from others, and through education. One study reported that self-efficacy increased significantly for socially withdrawn schoolchildren after participating in an intervention to improve this measure [56], which may indicate that self-efficacy is a resource for increasing the HRQOL among bullied children.

Our results highlight the importance of targeting self-efficacy beliefs as an intervention strategy with adolescents involved in bullying. Moreover, it indicates that improving self- efficacy and developing positive coping behaviours, could be effective in increasing adolescents HRQOL. School is an important setting for the promotion of social skills, and which may also enhance health. Adolescents spend most of their time in school, and the school setting is therefore a critical arena for interventions and social support.

### Strengths and limitations

Potential limitations must be considered when interpreting our results. Because it was a cross-sectional study, it is not possible to make causal inferences. Adolescents who were absent on the day of the study did not participate and we cannot assess whether participants and nonparticipants differed in any respect. These results might be regarded as representative of adolescents in one region of Norway, but we do not know whether they generalize to the rest of the country. However, the school system in Norway is fairly homogeneous, so the findings should be similar for the same age group in other regions. We used two widely used questions from Olweus to measure bullying; however, these are just single items, and the variables were dichotomized to never being bullied/bully other, versus bully/being bullied once or twice or more during the last 3 months. It is left to be determined how well being bullied/bully other once or twice cover the criteria “over time” in the definition of the concept.

Strengths of the study include the relatively large sample of adolescents (overall response rate 74%), which is considered satisfactory, and that we used well-validated questionnaires. We have studied the associations between GSE and bullying and HRQOL. In our discussion, we indicate that GSE may moderate the effect of bullying on HRQOL. However, this should be studied further by testing the mediating effects with a structural equation modelling approach.

## Conclusions

In this cross-sectional study, we found that being bullied and bullying others were both associated with lower HRQOL, and that higher GSE scores were associated with better HRQOL in adolescents. Self-efficacy contributed significantly to predict variation in HRQOL dimensions and may mediate the negative association between bullying and HRQOL. Assessing HRQOL among adolescents allows us to discover threats to their well-being and to become more aware of vulnerable subgroups. The relationship between GSE and HRQOL indicates that self-efficacy might be a resource for increasing HRQOL among adolescents. School is important for children’s social and emotional development, and an important arena for interventions. Our findings highlight the importance of targeting self-efficacy beliefs as an intervention strategy to improve GSE and HRQOL in adolescents involved in bullying. These findings need to be followed up in further studies; longitudinal studies could examine this relationship more thoroughly and determine the direction of the associations reported herein.

## Data Availability

The datasets analyzed as part of the current study are available from the corresponding author upon reasonable request.

## Abbreviations

GSE: General self-efficacy
HRQOL: Health-related quality of life
